# Findings from a survey of wildlife reintroduction practitioners

**DOI:** 10.12688/f1000research.3-29.v1

**Published:** 2014-01-29

**Authors:** Alexandra E. Sutton, Roel Lopez

**Affiliations:** 1 Department of Wildlife & Fisheries Sciences, Texas A&M University, College Station, 77843, USA

## Abstract

Wildlife reintroduction programs are a type of conservation initiative that seek to re-establish viable populations of a species in areas from which they have been extirpated or become extinct. Past efforts to improve the outcomes of reintroduction have focused heavily on overcoming ecological challenges, with little attention paid to the potential influence of leadership, management, and other aspects of reintroduction. This 2009 survey of reintroduction practitioners identified several key areas of leadership and management that may deserve further study, including: (i) the potential value of reintroduction partnerships for improving programmatic outcomes; (ii) the potential management value of autonomy vs. hierarchy in organizational structure; (iii) gaps in perceptions of success in reintroduction; and (iv) the need for improved evaluations of reintroduction programs and outcomes.

## Objectives

In the fight to preserve global biodiversity, conservationists and biologists must make use of every available tool and approach. Reintroductions are a type of triage initiative; a last-ditch intervention when every other effort to keep a species present within its historic range has failed. They are employed *only* in cases of significant biodiversity loss, and are subsequently operating under more dire conditions than any other type of conservation initiative. Regardless, they maintain a low success rate, estimated in the past 12 years between 26% and 32% ( Fischer & Lindemeyer, 2000; Jule *et al.*, 2008). Efforts to improve this success rate have focused heavily on improving biological knowledge as an avenue toward greater success. However, we suggest that another, overlooked, area of significant influence might lie in the human dimensions of reintroduction - specifically, the types of leadership and styles of management under which reintroduction programs are operated. Reliable data on reintroduction management is limited and restricted almost entirely to the gray (i.e. informally published) literature, with the exception of ( Clark & Westrum’s, 1989) paper on high-performance teams in wildlife conservation. This is unfortunate, as a slightly greater emphasis on the human dimensions of reintroduction would be to the benefit of both ecological and human communities. To that end, this survey is an exploratory effort to gain information about simple trends in reintroduction management and praxis, with the goal of informing future studies in this field.

## Methods

This survey was designed as an online-only, 47-question survey, presented via email between April and May 2009 and requiring approximately 20 minutes for completion. Emails of reintroduction practitioners were collected from the IUCN Reintroduction News online newsletter, the Reintroduction News Directory of Practitioners, and from the author contacts of reintroduction publications between 1999 and 2009, found through Google Scholar. There was no bias in participant selection relating to species, size or length of project, or budget. Invitations to participate in the survey were sent via email to 401 reintroduction practitioners worldwide.

## Survey design

The survey was designed subsequent to a case study of the leadership and management of the Sea Eagle Recovery Project, undertaken from May 2008 to August 2009 (Sutton, unpublished data). The six sections of the survey included two introductory demographic sections and four project-based sections, within which questions were designed based on observations made during the 2009 case study. These sections were: (i) About Your Project, (ii) About You and Your Position, (iii) About Organizational Structure, (iv) About Goal-Setting, Meetings and Evaluation, (v) About Public Relations and Outreach, and (vi) About Success and Performance. General trends and descriptive statistics were drawn from the data using Qualtrics website software (Qualtrics, 2009).

## Results

Sixty-eight (16.95%) invitees responded to the survey. An additional 40 (9.98%) responded to email invitations and stated that (a) they no longer worked in the field; (b) they had only conducted retrospective analyses of reintroduction and not participated in a program; or (c) they did not, for other reasons, wish to share their experiences. An additional 25 (6.23%) were not contactable by email (i.e. email addresses were outdated). The remaining 268 invitees (66.83%) did not respond. Reminders were sent to invitees at the two-week and one-month mark.

## Respondent demographics

**Figure 1.**
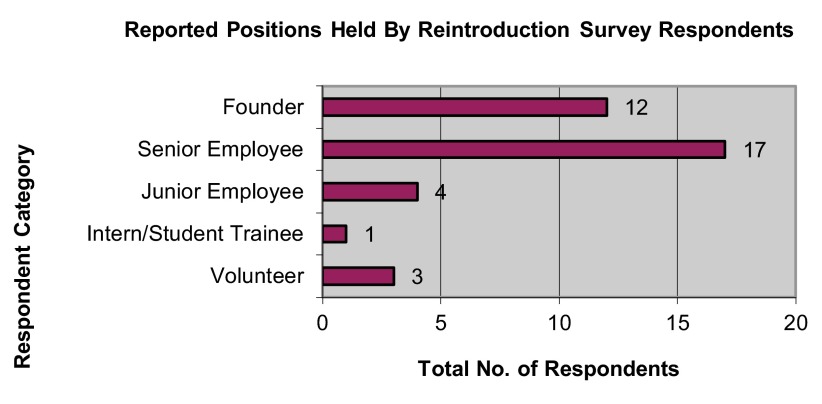
Reported positions held by reintroduction survey respondents.

**Figure 2.**
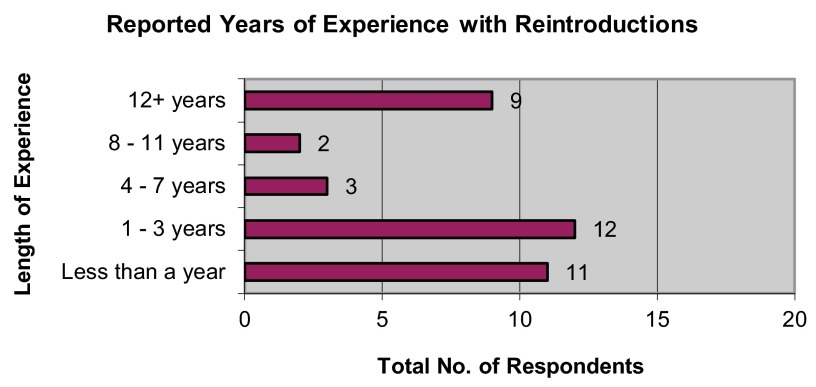
Reported years of experience with wildlife reintroductions among survey respondents.

Most respondents (45.95%) had served as senior employees or founders of reintroduction programs ( Figure 1), with the majority of respondents (62.16%) also reporting less than three years’ experience at the time they took on that role with the reintroduction program ( Figure 2).

## Reintroduction phases and lengths

Questions about phase length revealed four reintroduction phases: (1) planning, (2) approval, (3) action, and (4) monitoring. “Planning phase” referred to the period of time used to conceive and plan the reintroduction project. “Approval phase” referred to the period of time used to gain permission from government agencies or leading organizations to reintroduce the focal species. “Action phase” referred to the period of time during which animals were actually captured, captive-bred, raised, and released into the wild. “Monitoring phase” referred to the period of time during which reintroduced animals were monitored post-release.

**Figure 3.**
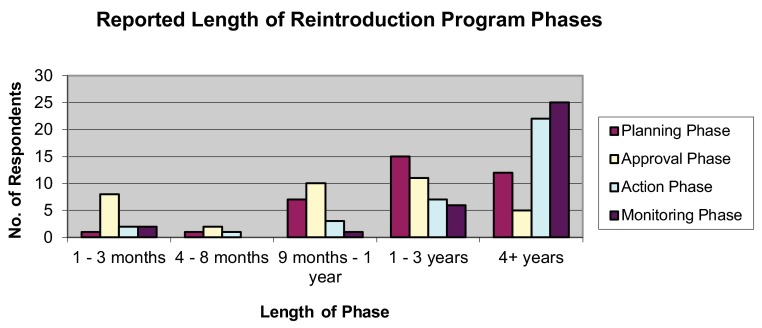
Reported length of reintroduction program phases.

Results indicated that planning phases most frequently took one to three years, while approval phases typically took nine months to one year. Both action and monitoring phases most commonly took more than four years ( Figure 3).

## Task supervision and organizational structure

**Figure 4.**
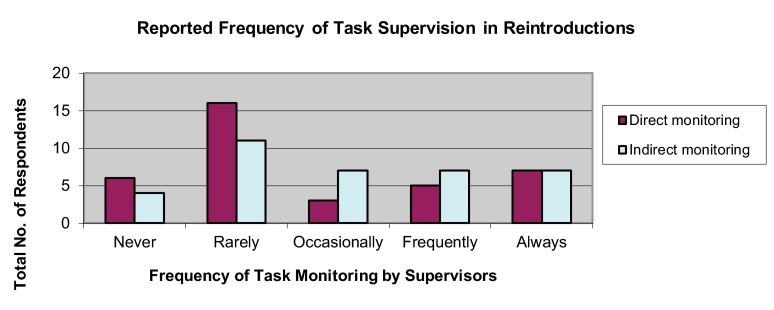
Reported frequency of task supervision in reintroduction programs.

**Figure 5.**
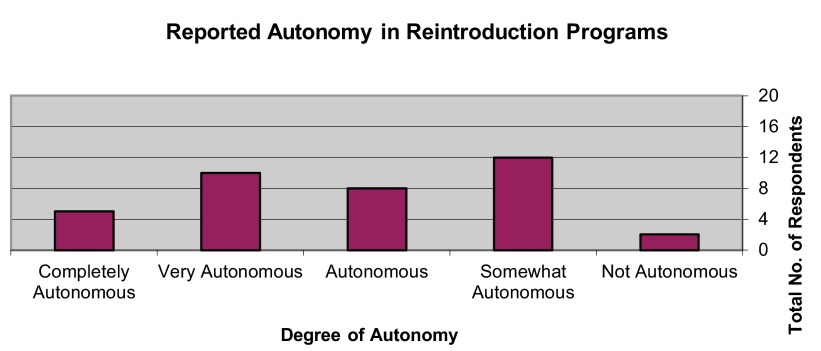
Self-assessed autonomy in reintroduction programs.

Respondents indicated that tasks were ‘rarely’ monitored, either directly (43.24%) or indirectly (30.56%), by supervisors ( Figure 4). Most respondents (32.43%) self-assessed their program as having been “somewhat autonomous”; however, a nearly-equivalent number self-assessed their program as having been “autonomous” (21.62%) or “very autonomous” (27.03%) ( Figure 5). Most respondents also indicated that their assigned tasks and responsibilities were “frequently” shared with coworkers (47.22%).

**Figure 6.**
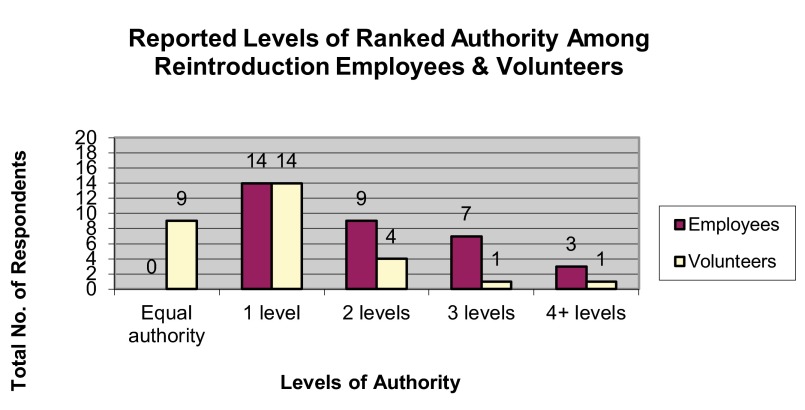
Reported levels of ranked authority among reintroduction employees and volunteers.

**Figure 7.**
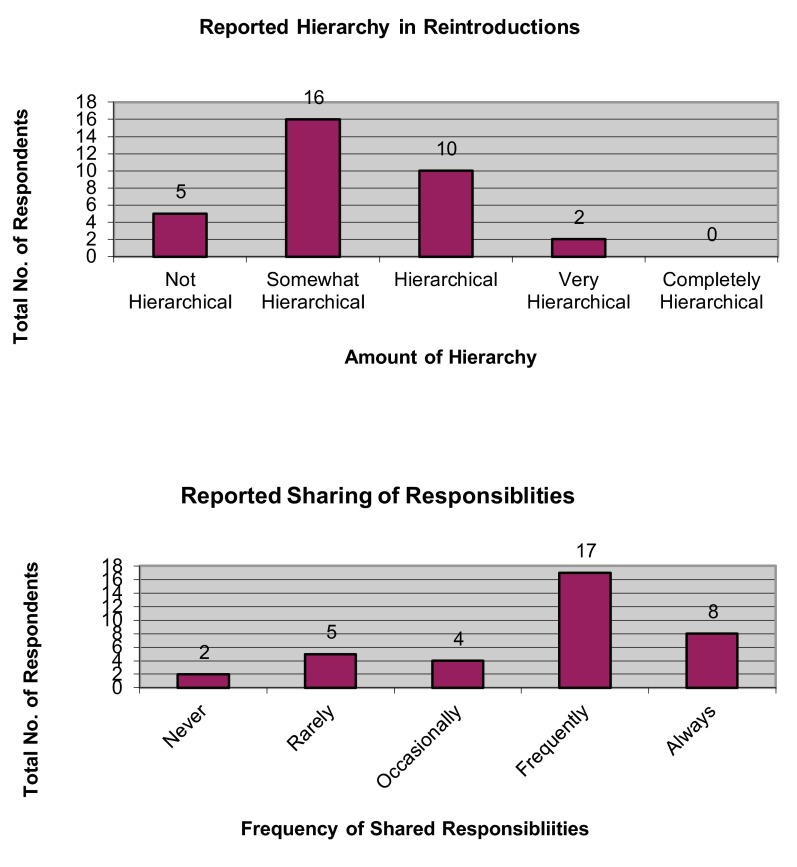
Self-assessed hierarchy in reintroduction programs.

Respondents most frequently reported two levels of authority existed between the most senior and most junior employee, and one level of authority existed between the most senior volunteer and most junior volunteer ( Figure 6). Most respondents (48.49%) self-assessed their projects as having been “somewhat hierarchical” ( Figure 7).

## Meetings and goal-setting

**Figure 8.**
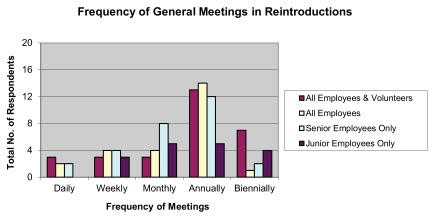
Reported frequency of general meetings in reintroduction programs.

**Figure 9.**
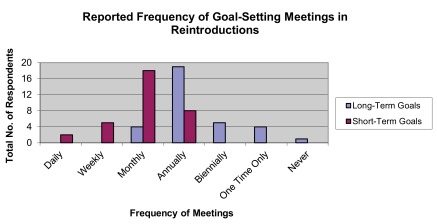
Reported frequency of goal-setting meetings in reintroductions.

The majority (56.00%) of all-staff, general meetings within reintroduction projects took place annually ( Figure 8). Most meetings that specifically aimed to establish, modify, or augment goals for the project were held annually to discuss long-term goals (57.58%) and monthly to discuss short-term goals (54.55%) ( Figure 9).

## Evaluation

**Figure 10.**
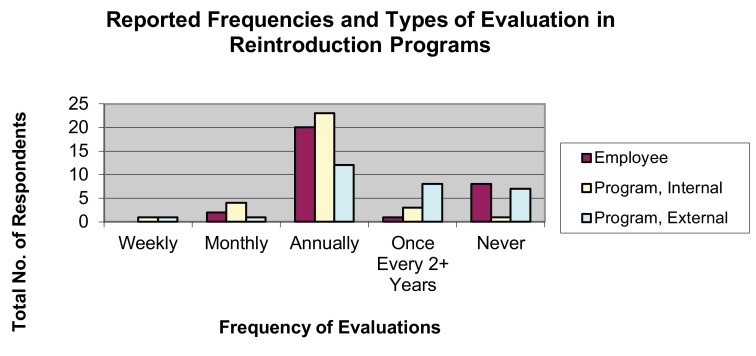
Reported frequencies and types of evaluation in wildlife reintroduction programs.

The majority of respondents reported evaluations of employee performance as an annual event (64.52%), as were evaluations of overall program outcomes, both by internal employees (71.88%) and external authorities (41.38%) ( Figure 10).

## Public relations and outreach

**Figure 11.**
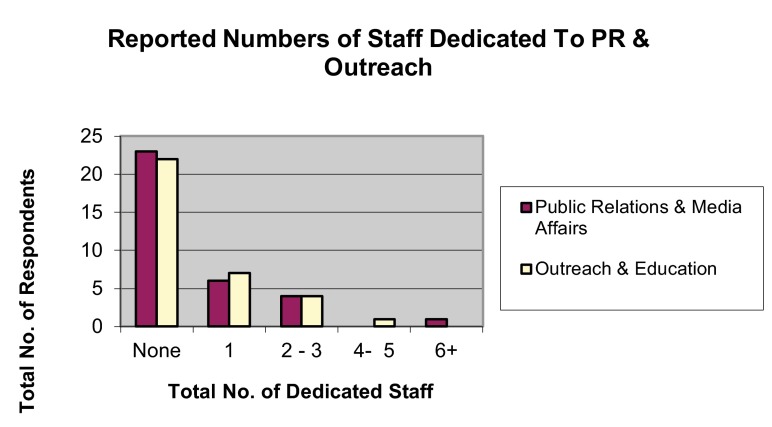
Reported numbers of staff dedicated to public relations and media affairs or public outreach and education.

Most programs had no staff dedicated solely to public relations/media affairs (67.65%) or public education and outreach (64.71%) ( Figure 11). Respondents indicated that projects were most likely to form partnerships with national wildlife organizations (77.42%) or local community groups (77.42%), and least likely to partner with corporations/businesses (43.75%) or other reintroduction programs (45.45%) ( Table 1).

**Table 1.** Reported partnerships of wildlife reintroduction programs.

Type of media	No partnerships	1–2	3–4	5–6	7+	Total projects reporting partnerships
Newspapers, magazines, or other forms of print media	9	13	8	0	2	23
Television/radio stations or other forms of audiovisual media	13	12	6	0	0	18
Websites, blogs, or other forms of internet media	13	13	5	0	1	19
Primary schools	13	6	3	0	8	17
Secondary schools	14	5	5	2	5	17
Colleges/Universities	10	12	3	3	2	20
International wildlife or conservation organizations	11	13	6	0	1	20
National wildlife or conservation organizations	7	14	9	1	1	24
Regional, local, or community organizations	7	10	7	3	4	24
Naturalist or local wildlife enthusiast organizations	11	11	4	3	3	21
Other reintroduction programs	18	10	3	1	1	15
Corporations or businesses	18	8	4	1	1	14

## Success and progress

**Figure 12.**
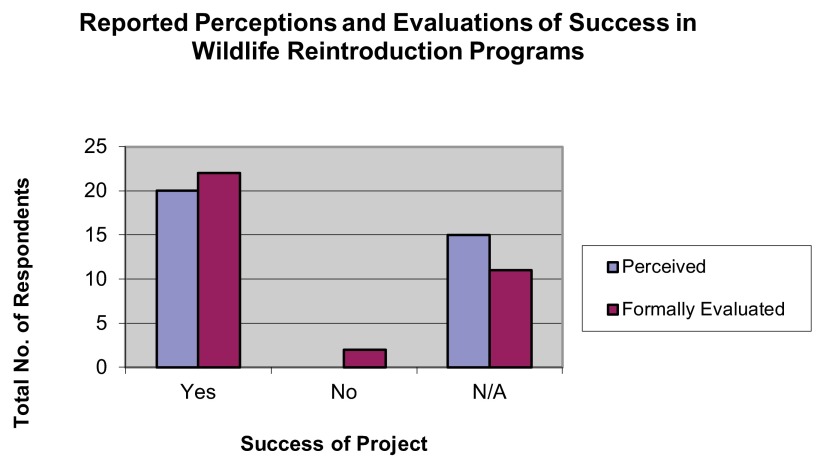
Self-assessed and evaluated success in wildlife reintroduction programs.

**Figure 13.**
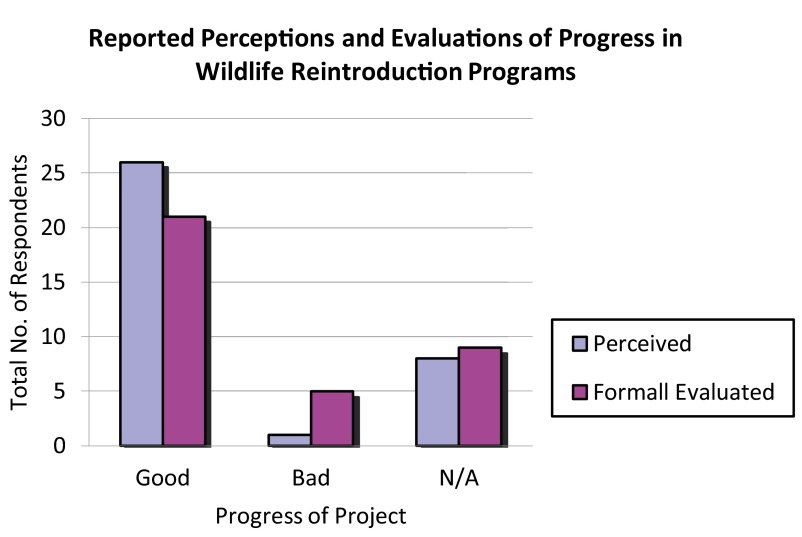
Self-assessed and evaluated progress in wildlife reintroduction programs.

Most respondents self-assessed their projects as having been a success (57.14%); most also reported a formal evaluation as having determined that their project had been a success (62.86%). A wide majority of respondents self-assessed their project as having “made good progress” (74.29%); most also reported that a formal evaluation had determined their reintroduction to have made good progress (60%) ( Figure 12, Figure 13).

Responses from a survey on leadership and management issues by wildlife reintroduction practitioners Responses of 68 wildlife reintroduction practitioners to a survey (Qualtrics, 2009) on management and leadership aspects of wildlife reintroduction programs. The survey was completed between April-May 2009.

## Discussion

The survey results show several trends in reintroduction management and reveal a multitude of gaps in knowledge and management practice. The clear gaps in knowledge, expertise, partnerships and evaluation yield a bevy of interesting questions for further study – and demonstrate the lack of a best practices management protocol in this field.

*Expertise gap*: despite respondents’ high-level roles as reintroduction founders or senior officers, they typically lacked reintroduction experience. Most respondents reported less than three years’ experience at the time they took on high-level roles; this is the same length of time typically required for planning and approval for a reintroduction, according to respondents’ reports. This overlap indicates that the majority of reintroduction founders and executives responding to this survey had never witnessed the full planning-approval-action-monitoring process of a reintroduction at the time they were placed in charge of one.

*Partnership and knowledge-sharing gap*: overall, respondents reported very limited engagement between their reintroduction and partner organizations of any type. Partnerships that were reported skewed heavily toward national wildlife or conservation organizations and national news outlets, and very few partnered with either businesses or other reintroduction programs. The former gap is a missed opportunity to engage corporate partners in conservation and build a stronger sponsorship base for local projects; the latter may indicate a tragic lack of connectivity between parallel projects, and hints at a likelihood of redundant work and “learned lessons” that go unshared.

*Evaluation gap*: the lack of established, recurrent evaluations conducted by external authorities was lamented by ( Kleiman *et al.*, 1999) in all areas of conservation, and is only too evident here. A trend toward frequent, informal, internal evaluations means that rigor is decreased; this decrease in rigor and shift toward informality has been recognized as a challenge to maintaining the value of program evaluation across all types of organizations ( Roch & McNall, 2007). This type of weaker evaluation can lead to a loss of accurate perceptions, as suggested by the gaps between respondents’ self-assessment of their programs’ success or progress and the results of formal evaluations.

Although the success-perception gap in our survey was not large (a 5.72% difference), the progress-perception gap was nearly triple (14.29%), and respondents reporting that they believed good progress had been made were common than those reporting that they believed success had been met (74.29% vs. 57.14%). This may suggest that respondents have a poor understanding of how to recognize markers of progress that lead to success – a problem that weak evaluation would only exacerbate.

## Summary

This survey, although preliminary, provided insight into several areas of conservation leadership and management that could be focal areas of future study. Understanding the gaps in expertise and evaluation, as well as the missed opportunities in partnership and knowledge-sharing, could be hugely beneficial in the future improvement of project management and reintroduction outcomes.

## References

[ref-1] Clark TW Westrum R: High-performance teams in wildlife conservation: a species reintroduction and recovery example. *Environ Manage.* 1989;13(6):663–670 10.1007/BF01868305

[ref-2] Fischer J Lindenmayer DB: An assessment of the published results of animal relocations. *Biol Conserv.* 2000;96(1):1–11 10.1016/S0006-3207(00)00048-3

[ref-3] Jule KR Leaver LA Lea SEG: The effects of captive experience on reintroduction survival in carnivores: A review and analysis. *Biol Conserv.* 2008;141(2):355–363 10.1016/j.biocon.2007.11.007

[ref-4] Kleiman DG: Reintroduction of captive mammals for conservation. *Bio Science.* 1989;39:152–161 Reference Source

[ref-5] Roch SG McNall LA: An investigation of factors influencing accountability and performance ratings. *J Psychol.* 2007;141(5):499–523 10.3200/JRLP.141.5.499-524 17933404

