# Supplementary material for: Findings from a survey of wildlife reintroduction practitioners
Source: F1000Res. 2014 Jan 29;3:29. [Version 1] doi: 10.12688/f1000research.3-29.v1 (PMC3945767; doi:10.12688/f1000research.3-29.v1)
Supplement: Peer review history

# Peer review: Referee response for version 1

**Published:** 2014-02-14

**Authors:** Mary Blair

**Affiliations:** 1 Center for Biodiversity and Conservation, American Museum of Natural History, New York, NY, USA

This short research paper presents the results of a survey of wildlife reintroduction practitioners. The paper is well-written and the results have relevance to future studies, but I suggest several revisions to further improve the paper.

In the Summary, the authors state that the results provide insight into areas of conservation leadership and management, although the article really only focuses on reintroduction programs specifically. There are many ongoing discussions in the wider conservation management arena about leadership and evaluation of success and it might be important to give the paper some more context in relation to those ongoing discussions (e.g. by referring to Black & Groombridge 2010 Conservation Biology, several publications related to the CMP Open Standards http://www.conservationmeasures.org/initiatives/standards-for-project-management, Manolis *et al. *2009 Conservation Biology).

Also, further discussion on what sorts of responses to other questions resulted in a perception of "success" in a given program would greatly strengthen the claims of the paper. To do this the authors could add a correlation analysis among variables, or, perhaps the authors could detail a few case examples?

Minor comments:

- In the Methods, it is stated that the survey was sent to practitioners worldwide, but of the 17% that responded, was there bias in terms of the countries respondents represented? A bit more elaboration on the potential of bias in the survey results (both geographic and other kinds of bias) would strengthen the paper.
   
- It would be very informative for future studies to include the survey instrument itself as an appendix.
   
- Table 1. It might be more informative if values in this table were represented as percentages or proportions to better illustrate the claims in the discussion section about how partnerships are "very limited" or "very few" with businesses or other reintroduction programs.

I have read this submission. I believe that I have an appropriate level of expertise to confirm that it is of an acceptable scientific standard.

# Peer review: Referee response for version 1

**Published:** 2014-02-13

**Authors:** Ryan Chisholm

**Affiliations:** 1 Department of Biological Science, National University of Singapore, Singapore, Singapore

The authors have conducted a survey of reintroduction practitioners and they have analysed the distribution of different project statistics, such as degree of autonomy of and frequency of meetings. They also present data on project success rates.

These data will no doubt be useful to people working in the field. I was surprised that the authors did not investigate whether any of the other factors, e.g., frequency of meetings, was correlated with project success. They obviously have the data to look at this. Why didn’t they?

Minor comments:

- p2: "Lindemeyer" -> "Lindenmayer"
- p2: Citation formatting: “Clark & Westrum’s (1989)”
- p7: Heading of column 2 in Table 1 could just be “0”, because otherwise it could be read as “number of partnerships”.
- p8, Figure 13: “Formall Evaluated” -> “Formally Evaluated”
- p8: Citation formatting again: “Kleiman et al. (1999)”

I have read this submission. I believe that I have an appropriate level of expertise to confirm that it is of an acceptable scientific standard, however I have significant reservations, as outlined above.

# Peer review: Referee response for version 1

**Published:** 2014-02-13

**Authors:** Gary Luck

**Affiliations:** 1 Department of Wildlife Ecology and Management, Charles Sturt University, Albury, NSW, Australia

**General comments**

I agree with the authors that much of the assessment of reintroduction success is focused on the ecological aspects of reintroduction programs, with little attention paid to how humans manage reintroduction programs. I agree also that some of these management aspects can be critical to program success, although I think the authors could have elaborated on this in more detail in the Introduction (Objectives) section. A survey of wildlife practitioners involved in reintroduction programs is therefore a useful addition to the literature.

The study employs a fairly basic survey instrument delivered via email. More information on the survey instrument (design, types of questions, justification for questions included etc.) could have been provided, including a copy of the survey published as supporting information. The response rate to the survey was low, and I wonder if this introduced any biases to the results? Finally, the Discussion doesn’t really elaborate on how the trends in the responses might actually impact on the success rate of reintroduction programs. Would greater expertise lead to more success? How much expertise might be needed and in what areas specifically? Would more partnerships with corporate bodies actually improve reintroduction success? Why?

 

**Specific comments**

 

**Objectives**:

- For the sentence ‘ *Regardless, they maintain a low success rate, estimated in the past 12 years between 26% and 32%*’ it would be instructive to know how the cited authors measured ‘success rate’.
- This section could include a more detailed argument regarding why ‘types of leadership’ or ‘styles of management’ are likely to be so important to reintroduction success.

**Methods**:

- Change ‘ *Emails of reintroduction practitioners…*’ to ‘Email addresses of…’ to avoid confusion.
- ‘ *There was no bias in participant selection relating to species, size or length of project, or budget*.’ - Were practitioners asked about these things? It would have been instructive to know the spread of responses (e.g. types of species dealt with).

**Survey design:**

- It would be useful to include more information about survey design including a copy of the actual survey published as supporting information. Additional information could include details of question design and type, and justification for inclusion.

**Results:**

- Did the low response rate lead to any biases in the results? 

**Task supervision and organizational structure:**

- Please define ‘autonomous’ in this context.

**Success and progress:**

- Again, a short definition of ‘success’ would be useful here. Was a definition included in the questionnaire or were practitioners just asked something like ‘Was your project successful?’ and just left to self-define the meaning of ‘success’?

**Discussion**

- A broader discussion of how specific results might influence reintroduction program success would be highly beneficial and help guide future research in this area. 

I have read this submission. I believe that I have an appropriate level of expertise to confirm that it is of an acceptable scientific standard.

# Peer review: Referee response for version 1

**Published:** 2014-02-05

**Authors:** David Norton

**Affiliations:** 1 New Zealand School of Forestry, College of Engineering, University of Canterbury, Christchurch, New Zealand

This is an interesting short note that addresses a useful question. While it is true that environmental factors play a key role in limiting the success of reintroduction programmes, management and leadership factors could also be an important issue. This note sought information on this issue through a survey of people involved in reintroduction programmes. I think the two key conclusions from this survey are useful, that there is a lack of long-term experience amongst those undertaking reintroduction programmes, and that there is a lack of formal evaluations of these programmes. This latter point also links to the observation that there is a lack of linkages between different organisations and different reintroduction programmes.

There were, however, some issues that I felt did require further attention: I was unclear what the focus of the survey was - was it just in the USA or was it wider? This should be clarified. Was there a bias in the responses in that perhaps only those that had been involved with more successful reintroduction programmes responded? The survey results indicate success rates of around 60% which is much higher than the 26-32% cited in the Objectives section. This should be discussed further. I would also have liked to have seen more discussion in the conclusions on how the results of this survey might be used. How might these results be taken up and used to improve future re-introduction programmes? I also felt that the paper had too many figures and wondered if some of these could be removed with just the key results presented in the text (or perhaps tabulated). I thought the abstract was a good summary of the article, but was unclear what the difference is between extirpation and extinction.

I have read this submission. I believe that I have an appropriate level of expertise to confirm that it is of an acceptable scientific standard, however I have significant reservations, as outlined above.
